# Are HFpEF and HFmrEF So Different? The Need to Understand Distinct Phenotypes

**DOI:** 10.3389/fcvm.2021.676658

**Published:** 2021-05-21

**Authors:** Alberto Palazzuoli, Matteo Beltrami

**Affiliations:** ^1^Cardiovascular Diseases Unit, Department of Medical Sciences, Le Scotte Hospital University of Siena, Siena, Italy; ^2^Cardiology Unit, San Giovanni di Dio Hospital, Florence, Italy

**Keywords:** ejection fraction, heart failure with mid-range ejection fraction, phenotype, biomarkers, systolic function

## Abstract

Traditionally, patients with heart failure (HF) are divided according to ejection fraction (EF) threshold more or <50%. In 2016, the ESC guidelines introduced a new subgroup of HF patients including those subjects with EF ranging between 40 and 49% called heart failure with midrange EF (HFmrEF). This group is poorly represented in clinical trials, and it includes both patients with previous HFrEF having a good response to therapy and subjects with initial preserved EF appearance in which systolic function has been impaired. The categorization according to EF has recently been questioned because this variable is not really a representative of the myocardial contractile function and it could vary in relation to different hemodynamic conditions. Therefore, EF could significantly change over a short-term period and its measurement depends on the scan time course. Finally, although EF is widely recognized and measured worldwide, it has significant interobserver variability even in the most accredited echo laboratories. These assumptions imply that the same patient evaluated in different periods or by different physicians could be classified as HFmrEF or HFpEF. Thus, the two HF subtypes probably subtend different responses to the underlying pathophysiological mechanisms. Similarly, the adaptation to hemodynamic stimuli and to metabolic alterations could be different for different HF stages and periods. In this review, we analyze similarities and dissimilarities and we hypothesize that clinical and morphological characteristics of the two syndromes are not so discordant.

## Introduction

Despite the last ESC guidelines introducing a new category for heart failure (HF) classification including those patients with mild ejection fraction (EF) reduction ranging from 40 to 49%, this subtype is still underdetermined and poorly represented in most clinical trials (1). Current gaps arise from the recent introduction of this HF class and the indeterminate profile between heart failure with preserved ejection fraction (HFpEF) and heart failure with reduced ejection fraction (HFrEF) that probably account for different phenotypes. Indeed, the ESC classification is an attempt to identify specific biological and pathophysiological mechanisms in subjects with clinical manifestations typical for HF, increased natriuretic peptides, and moderate structural cardiac dysfunction (2). Perhaps, HFmrEF is a mixed model related to the intermediate clinical profile between HFpEF and HFrEF, encompassing patients with phenotypic and clinical characteristics typical for both reduced and preserved EF (3). Indeed, few studies analyzing HFmrEF subtypes demonstrated some discrepancies in terms of comorbidity and etiology (4). However, the simple categorization based only on EF keeps some weaknesses related to the intrinsic limitation of EF, its change over a time period, and the natural history of HF. Therefore, EF measurement depends on several intrinsic variables such as preload and afterload, heart rate, stable or unstable condition, myocardial contractile forces, and presence of valve disease (5). Of note, both American and recent Australian HF guidelines preferred to maintain the traditional classification of HFpEF and HFrEF based on EF cutoff of 50%, so as not to create a misunderstanding and overlap in HF nomenclature (6, 7). Thus, the attempt to classify HF population based on simple EF categorization is probably inappropriate, and the identification of a unique profile for HFmrEF is a pending issue (8). Classification of HF patients into the whole spectrum of different phenotypes within EF assessment remains a challenge for future research.

## Clinical Characteristics of HFmrEF

HFmrEF is an heterogeneous group poorly characterized in terms of baseline characteristics, clinical presentation, and outcome (4). Most of the data came from subanalysis investigating the features of patients with HFpEF and borderline EF or from HFrEF trials analyzing patients with recovered systolic function (9, 10). In order to bypass this gap, recent studies have focused on the HFmrEF distribution and risk profile investigation; unfortunately, most of them are single center with unrepresentative sample size and with incomplete standardized diagnostic criteria based only on EF cutoff. Clinical characteristics, cardiovascular (CV) risk profile, extracardiac comorbidities, and echocardiographic features are often neglected, leading to a further confusion in HFmrEF recognition and some discrepancies between studies. Of note, most of the data can be extrapolated by larger clinical trials with a relevant follow-up period including this category. The CHARM preserved study that included patients with EF >40% showed that most patients with mildly reduced EF were females with intermediate mean age values and hypertension prevalence between HFrEF and HFpEF (11). Therefore, HFmrEF has a similar prevalence to coronary artery disease (CAD) and atrial fibrillation (AF) compared with HFrEF, whereas creatinine values and NYHA class distribution were intermediate between HFrEF and HFpEF. Despite different clinical characteristics, the study revealed a reduced trend of HF-related hospitalization and death for CV causes with respect to HFrEF.

The restrospective analysis of the DIG trial demonstrated that HFmrEF resembled patients with HFrEF in terms of similar mean age, sex, and ischemic etiology (12). In the TOPCAT trial involving patients with mean EF above 45%, mean age and female prevalence were higher in those with mildly reduced EF, hypertension was higher in HFmrEF, whereas other comorbidities such as chronic kidney disease (CKD), CAD, AF, and diabetes were similar between groups (13). Interestingly, a Korean registry revealed different prevalence rates of AF that tend to increase according to EF values with different occurrences in reduced (29%), midrange (40%), and preserved (45%). Additionally, AF has a negative prognostic impact only in HFpEF (14).

The ESC observational registry confirmed that patients affected by HFmrEF resembled the HFpEF group in some features including age, female prevalence, and hypertension. However, CAD prevalence was more similar to the HFrEF group. Mortality rate at 1 year significantly differed between HFpEF and HFmrEF (6.3 vs. 7.6%, respectively) (15). A validated analysis using MAGGIC score including a wide range of cardiac and extracardiac and demographic characteristics demonstrated that an increased burden of extracardiac diseases in those with higher EF with a significant prevalence of lung diseases increased body mass index and diabetes (16). Accordingly, a Japanese registry confirmed an intermediate profile of HFmrEF patients supposing that the current condition may be a transitional status between HFpEF and HFrEF (17). In a recent Swedish registry analysis comparing three common comorbidities such as AF diabetes and CKD, HFmrEF revealed an intermediate prevalence of CKD and AF, whereas diabetes was similarly expressed in all HF groups (18). Finally, the combined analysis of PARADIGM and PARAGON confirmed an intermediate range regarding age, female sex, body mass, natriuretic peptides, and hypertension, whereas history of myocardial infarction resembled HFrEF (19).

Current findings are related to chronic HF conditions, but acute patients presenting with HFmrEF are less extensively investigated: in the ALARM HF trial that stratified patients for EF tertiles, majority of the patients were male with consistent prevalence of older age more than 75 years, obesity, hypertension, and dyslipidemia; with intermediate prevalence of CAD; and lower prevalence of CKD with respect to HFrEF. No differences were observed in terms of anemia, lung diseases, vascular diseases, and liver disease (20). The main causes of hospitalization were acute coronary syndrome (ACS) in 38.6%, arrhythmias in 25.8%, and valvular disease in 15.4%. Clinical presentation differed between HFmrEF and HFrEF in terms of less peripheral edema, jugular vein distention, and prevalence of cold extremities. Current findings considerably differ from those observed in the DIG in which HFmrEF had less prevalence of orthopnea and additional cardiac sound compared with HFrEF (12). Conversely, exertional dyspnea, dyspnea at rest, and peripheral edema were similar in both HFrEF and HFmrEF (Table 1).

**Table 1:** Clinical trials describing prevalent risk factors, comorbidities, and causes of HFmrEF.

**Clinical Trial**	**Type of study**	**Population enrolled**	**NYHA Class**	**Outcome**
CHARM preserved 2018	*Post-hoc* analysis including 1,322 pt	Mean age 65 year, mean EF 44%, 30% females, BMI 27.8, 67% CAD, 56% hypertension, 25% AF	57% II 41% III	HF hospitalization reduction (HR 0.48) Mortality reduction per year (HR 0.76)
DIG trial 2018	Retrospective analysis including 1,195 pt	Mean age 64.5 year, mean EF 43%, females 29%, BMI 27.7, previous MI 63%, hypertension 53%, AF not reported	3 % I 62% II 20 % III	Composite endpoint HF-hospitalization /mortality HR 0.83
TOPCAT trial 2016	Retrospective analysis including 520 pt	Mean age 66 years, mean EF <50%, females 36.5%,BMI 31.5, previous MI 44%, hypertension 86%, AF not reported, diabetes 29%	3% I 61% II 35% III	CV death per 100 patient-years HR 4.1HF hospitalization per 100 patient-years HR 7.2
Korean HF registry 2020	Prospective observational study including 875 acute pt	Mean age 69 years, Mean EF 49%, females 45%, BMI not reported, CAD 29%, hypertension 59%, AF 27% AF, diabetes 36%	18% II 41%III 41%IV	Composite end point for all cause mortality and readmission HR 1.14
ESC -HF registry 2017	Observational research program of 2,212 pt	Mean age 64 years; females 31%, BMI 28.6, previous CAD 42%, hypertension 10%, AF 22%, Diabetes 30.5%, CKD 16,5%	82% I/II 18% III/ IV	Mortality at one year 7.6% in HFmEF vs. 6.3% in HFpEF and 8.8% in HFrEF
chart-2 investigators 2017	Japanese registry including 596 pt	Mean age 69 years, mean EF 45%, females 28%, BMI 23, previous MI 53%, hypertension 90%, AF 43.5%, diabetes 36%, CKD not reported	18.5% I 70% II 11% III	HFmrEF patients had intermediate incidences of all-cause death, and CV admission between HFpEF and HFrEF; 44% transitioned from HFmEF to HFpEF
Swedish HF registry 2019	Categorial analysis including 8,942 pt	Mean age 74 years, mean EF 44%, Females 38%, BMI 28, previous CAD 62%, hypertension 71%, AF 27%, diabetes 24%, CKD 46%	16% I 48% II 37%III 4% IV	HFmrEF had lowest crude risk of all CV and HF events, but it was intermediate between HFpEF and HFrEF for the crude risk of non-CV events
PARAGON and PARADIGM combined data matched for EF categories	*Post-hoc* analysis including 1,427 pt	Mean age 71 years, mean EF 48%, females 40%, previous MI 32%, hypertension 94%, AF 34%, diabetes 44%	3%I 76% II 21% III	Total heart failure hospitalization and CV death 0.81 in HFmEF vs. 1.06 in HFpEF
ALARM-HF prospective trial 2017	Multicenter survey including 811 acute pt	Mean age not reported, Mean EF 44%, females 35%, history of CAD 29%, hypertension 76%,AF 42 %, diabetes 46%	9.8% I 8.3 %II 47% III 35 %IV	Mortality in HFmEF was similar in HFmEF and HFpEF (HR 1.02 vs. 0.97)

*AF, atrial fibrillation; BMI, body mass index; CAD, coronary artery disease; CKD, chronic kidney disease; EF, ejection fraction; MI, myocardial infarction*.

Aside from clinical characteristics and presentation, a few discrepancies are related to the outcome and mode of death of this group: although some studies reported a similar mortality rate independently of EF, some authors revealed an intermediate clinical profile and risk between HFpEF and HFrEF, and there is a general agreement in considering the outcome of HFmrEF much more similar to HFpEF (21, 22). Despite that CV events are considerably more in HFrEF, prognosis in those with HFmrEF is more strictly related to non-CV events and this tends to balance the overall mortality rate (23).

## Laboratory Profile of HFmrEF

The division of HF across the EF spectrum comprises different biochemical and neurohormonal profiles that help to explain the neutral effects of interventional trials testing neurohormonal antagonism in HFpEF. N-terminal pro-B-type natriuretic peptide (NT-proBNP), plasma renin activity (PRA), aldosterone, and norepinephrine are increased in a substantial proportion of patients with HFpEF and HFmrEF with the same levels between the above groups and with lower levels when compared with HFrEF. Vergaro et al. demonstrate that 10% of HFpEF patients had elevated PRA, aldosterone, and norepinephrine vs. 8% in HFmrEF and 21% in HFrEF. The prognosis of HF patients seems to correlate with the number of neurohormones elevated, and different degrees of neurohormonal activation are evident across the whole EF spectrum, suggesting a positive effect of renin–angiotensin–aldosterone system inhibitors (RAASi) and adrenergic antagonists in patients with a significant increase of the aforementioned biomarkers (24). A specific biomarker analysis from the Swedish Heart Failure Registry revealed similar NT-proBNP levels in HFmrEF and HFpEF, but significantly lower to HFrEF. However, body mass index (BMI), CKD, diabetes, hypertension, and heart rate significantly influence NT-proBNP levels. Nevertheless, NT-proBNP shows a greater prognostic in HFmrEF and may be a useful tool for diagnosis and stratification of CV risk (25).

The PROTECT trial analyzes several biomarkers of cardiac stretch and inflammation in acute HF setting. The network analysis demonstrates that inflammation is the main reason of interactions between biomarkers in HFpEF [e.g., galectin-3 (Gal-3) or C-reactive protein (CRP)], whereas in HFrEF, biomarker interactions are mostly related to cardiac stretch [e.g., NT-proBNP or high-sensitivity troponin (hs-TnT)]. Patients with acute HFmrEF show an intermediate profile between those of HFrEF and HFpEF. A small proportion of patients enrolled in the HFmrEF group are considered with “recovered LVEF,” and interestingly, NT-proBNP, Gal-3, and hs-TnT are lower than in patients with persistent EF reduction, suggesting a different biomarker profile in this phenotype. However, in both HFpEF and HFmrEF, inflammatory markers at admission are both predictive for all-cause mortality and rehospitalization (26). Similarly, the Singapore Heart Failure Outcomes and Phenotypes (SHOP) study show intermediate values of hs-TnT with significant increased values compared with HFpEF (27).

The study with better laboratory and biological profile investigation is currently the HOMAGE trial; unfortunately, the laboratory analysis is limited to patients with a high risk of HF occurrence, history of CAD, and evidence of borderline EF dysfunction above 45%, but without specific signs and symptoms suggestive of HF (28). Patients with EF below the normal range experienced raised plasma B-type natriuretic peptides (BNP) and fibrosis biomarkers, whereas an increased level of inflammatory and collagen markers has been recruited in those with significant cardiac hypertrophy. Spironolactone significantly reduced natriuretic peptides, biomarkers of collagen, and inflammation (29).

Another study reported the bioprofile and the bioprognostication of several biomarkers of neurohormonal activation, extracellular matrix, inflammation, oxidative stress, and myocardial injury in patients with HFmrEF. Cystatin-C levels were significantly lower in patients with HFmrEF when compared with patients with HFpEF. The results of soluble suppression of tumorigenicity (sST2) levels, a member of the interleukin family, in HFmrEF patients are controversial which may be due to confounding factors such as race, HF congestion status, population enrolled, and disease time course. However, sST2 levels correlate with advanced NYHA class, pulmonary arterial systolic pressure, hs-CRP, cTnT, NT-proBNP, and the high frequency of diuretics use. Conversely, Gal-3 seems to be lower in HFmrEF than in HFpEF, showing the highest prognostic capability in the latter group (30).

In a selective group of patients with type 2 diabetes mellitus and HFpEF or HFmrEF, C-terminal propeptide of procollagen type I (PICP) and N-terminal propeptide of procollagen type III (PIIINP) are significantly increased in patients with HFmrEF compared with those with HFpEF. Glucometabolic impairment stimulated fibroblast proliferation and activated transcription and secretion of extracellular matrix proteins. The changes found in both markers of fibrosis may suggest a shift in balance toward type I collagen synthesis in HFmrEF compared with HFpEF in diabetic patients (31). Finally, we could assume that analyzing the various biomarker profiles in all HF population does not take into account the several mechanisms that are shared across the entire EF range. Some processes are more relevant at the extremities (HFrEF myocyte death vs. HFpEF inflammation or fibrosis), and in this spectrum, HFmrEF represents a continuum without a predominant underlying pathophysiology (32, 33). In this era in which a new precision phenotype is emerging in patients with HF, knowledge of different pathophysiologic pathways and of the laboratory profile of each patient may contribute to therapeutic decision and prognostic stratification (Table 2).

**Table 2:** Biomarker characteristics and differences existing between HFmrEF and HFpEF.

**Biomarkers profile in HF patients according to EF spectrum**		
**Diagnosis**	**HFpEF**	**HFmEF**
	NT-proBNP↑	NT-proBNP↑↑
	hs-TnT ↑	hs-TnT ↑↑↑
	Plasma renin activity ↑	Plasma renin activity ↑
	Aldosterone ↑↑	Aldosterone ↑
	Norepinephrine ↑	Norepinephrine ↑
	hs-CRP ↑↑	hs-CRP ↑
	Cystatin-C ↑↑	Cystatin-C ↑
	Galectin-3 ↑↑	Galectin-3 ↑
	Neprilysin ↑	Neprilysin ↑↑
	ST2 ↑↑	ST2 ↑
	PICP ↑	PICP ↑↑
	PIIINP ↑	PIIINP ↑↑
Prognosis	NT-proBNP+	NT-proBNP+++
	hs-TnT +	hs-TnT ++
	hs-CRP ++	hs-CRP +
	Cystatin-C +	Cystatin-C +
	Galectin-3 +	Galectin-3 +
	Neprilysin +	Neprilysin +
	ST2 ++	ST2 +

*EF, Ejection Fraction; NTproBNP, N-terminal pro-brain natriuretic peptide; hs-TnT, high-sensitivity troponin T; hs-CRP, high-sensitivity C-reactive protein; ST2, soluble suppression of tumorigenicity 2; PICP, C-terminal propeptide of procollagen type I; PIIINP, N-terminal propeptide of procollagen type III; ↑, diagnostic accuracy to detect heart failure subtypes; +, prognostic significance for each biomarker*.

## Limitations Related to EF Assessment

The EF threshold constitutes the hallmark variable for HF subtype identification and categorization. Notably, EF offers some advantages related to immediate comprehension, short scan time, and feasibility—not requiring specific expertise (34, 35). Therefore, EF can be calculated easily by using echocardiographic application, and it can be assessed visually even without a specific background. Moreover, EF provides the basis for structural and functional phenotype classification, and it is universally accepted in clinical practice and in study research (36). Beyond these features, EF assessment and related HF classification has demonstrated several gaps due to mechanistic, methodological, and hemodynamic pitfalls that do not really describe the true contractile ventricular function and pressure–volume relationship status (37).

**Figure 1:**
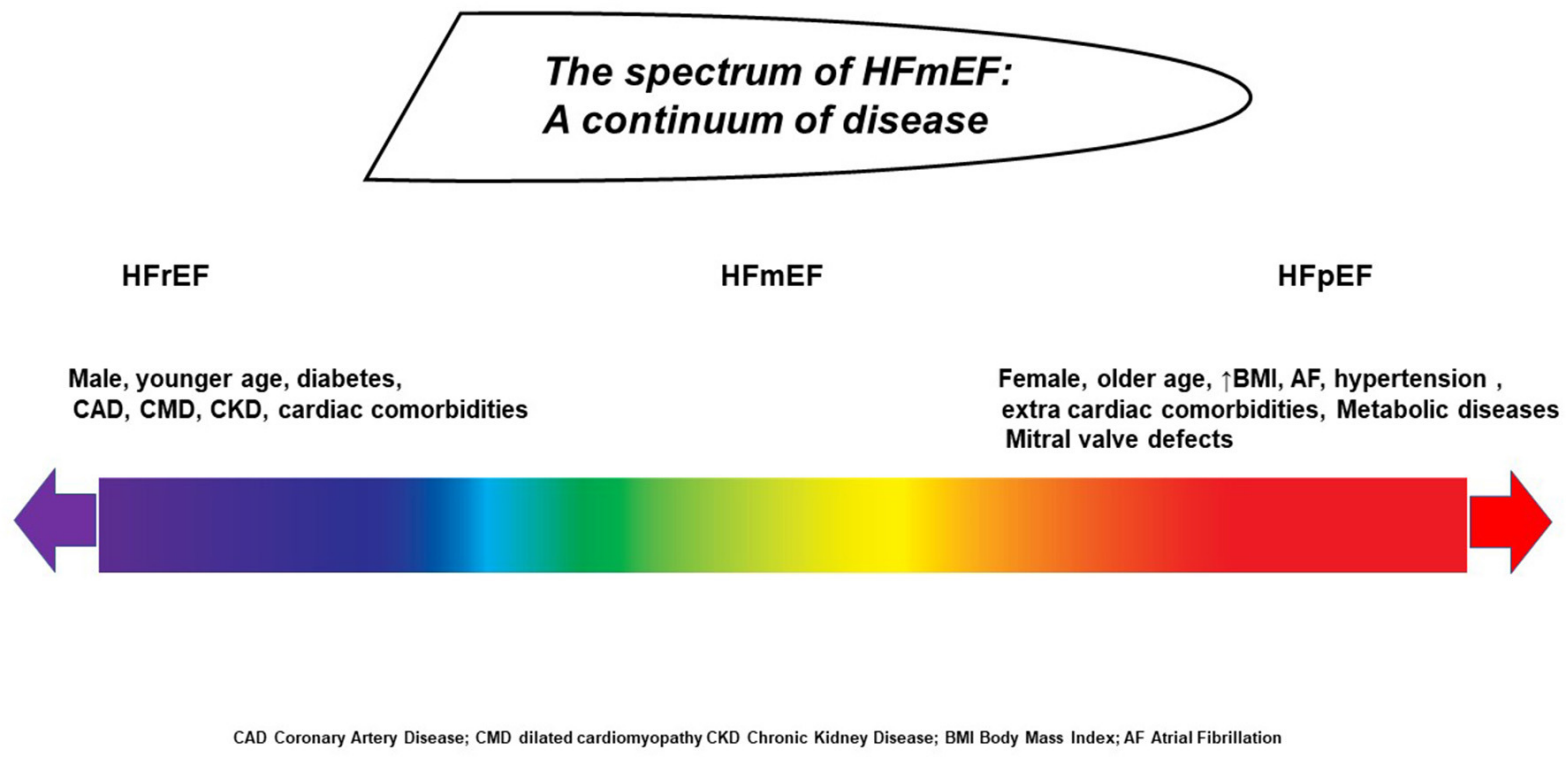
The spectrum of patients with HF ranging from severe ejection fraction reduction to preserved function, according to baseline phenotype, disease time course, response to therapy, and loading conditions.

EF is sensitive to sudden changes in preload and afterload forces, and sudden elevation in systemic blood pressure or vascular stiffness could impair the measurement. Conversely, a reduction in preload, causing a decrease in the atrioventricular blood afflux, makes the LV emptying more efficacious by a reduction of parietal strain forces (5, 38). In the presence of a valve defect, EF may be over- or underestimated: in case of significant mitral regurgitation, EF will be augmented because of the reduced workload during cardiac contraction. Otherwise, during aortic stenosis, an increase of afterload occurs along with a delay in outflow time peak and consequent EF reduction (39). Other factors such as intrinsic myocyte forces, distension capacity, cronotropic incompetence, ventriculo-arterial coupling, and pressure–volume curve adaptation during exercise are all potential confounders for EF estimation (40). Chronic heart rate increase or decrease could underestimate or overestimate the values, respectively. Similarly, sympathetic activity or vagal stimulation and other systemic conditions such as anemia, thyroid dysfunction, and endocrine and metabolic alterations are all features that could potentially influence EF assessment. Behind these features, the HFmrEF subtype can be derived from patients with a previous and more severe EF reduction having a good response to therapy as well as from subjects with initial preserved EF experiencing initial systolic dysfunction (41). All these concerns highlight the need for a more comprehensive approach including environmental, social, genetic, and metabolomic factors in order to better characterize this syndrome. Therefore, patients' history, associated risk factors, comorbidities, body size conformation, and response to therapy should be taken into account beyond the simple EF calculation (42). The real challenge is to concretize and combine several epidemiological, biohumoral, mechanistic, and cardiac functional data across a spectrum of different phenotypes in which each subject has a specific HF onset, development, and pathophysiological pathways (43). Indeed, the population included in the HFmrEF category is extremely variable, encompassing patients with different disease triggers, demographic characteristics, associated diseases, and mortality risks (Figure 1).

EF is usually measured by echocardiography; unfortunately, the interobserver variability even in accredited echo laboratories ranges from 5 to 18% with broader limits for less experienced physicians (44). Thus, the current ESC cutoff distinguishing HFpEF (for patients with EF >50%) from midrange EF (for patients with EF between 40 and 49%) makes this classification hard to distinguish, and it could reveal significant misclassification depending on the laboratory site and the physician's experience and skills. Finally, EF is erroneously considered a measurement of systolic function, but it is just an estimation of radial function. EF is not a reliable measurement of longitudinal and torsional contraction although the whole systolic function results from all three variables. This reflects the different course and geometrical alignment of myocardial fibers that are not homogeneous inside the myocardial wall and in the different cardiac sites varying from basal to apical segments (45, 46). Accordingly, several studies that included patients with preserved EF showed significant longitudinal global function impairment, despite an apparently normal systolic function (47, 48). These difficulties represent a challenge for future investigation and could be overcome with the extensional use of cardiac magnetic resonance and 3D echo by the construction of a specific software algorithm.

**Figure 2:**
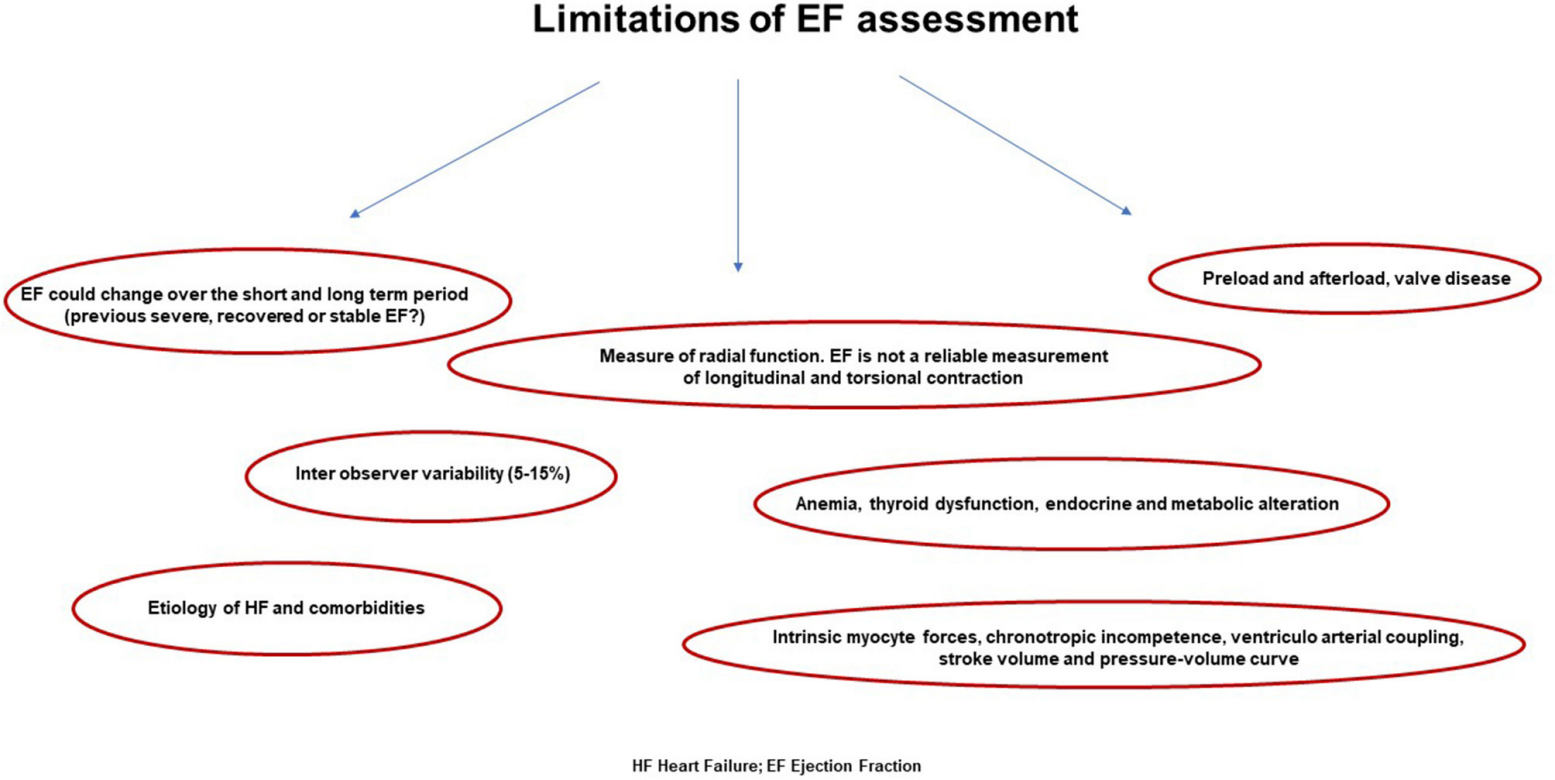
Main weakness in ejection fraction calculation that does not comprise several features revealing the real systolic function of the left ventricle.

Although it is not strictly related to the real forward flow, EF is erroneously considered as an indicator for LV remodeling. Indeed, an enlargement of diastolic dimension works as a compensatory factor in order to maintain an adequate stroke volume even during the occurrence of dilated systolic volume (46, 49). Conversely, in case of concentric remodeling, the stroke volume may be maintained although end diastolic volume is within the normal range and the ratio to systolic volume has altered. Notably, EF is inversely related to systolic volume but poorly related to stroke volume; thus, it is a mirror of systolic dysfunction in eccentric remodeling, whereas in concentric remodeling, it does not reflect effective contractile decline (50, 51) (Figure 2). For all these reasons, EF cannot be considered the only one reference of systolic function and may be contextualized into different cardiac remodeling, loading conditions, filling pressure, and hemodynamic status.

## Conclusions

HFmrEF represents a mixed model between HFpEF and HFrEF. Demographic, structural, and laboratory data resembled HFpEF, whereas the CAD prevalence and the response to management are likely associated with HFrEF. Because of distinct phenotype, HFmrEF might be differentiated from other HF subgroups, but it deserves further research investigating cardiac and extracardiac diseases influencing its appearance. Therefore, the simple HFmrEF categorization based only on EF cutoff appears misleading, and it should be contextualized with other variables comprising both CV risk factors and detailed cardiac morphological assessment.

## Author Contributions

All authors participated in the manuscript draft review and design. The author warrants that his/her contribution is original and that he/she has full power to make this grant. The author signs for and accepts responsibility for releasing this material on behalf of any and all co-authors. The copyright transfer covers the exclusive right and license to reproduce, publish, distribute, and archive the article in all forms and media of expression now known or developed in the future, including reprints, translations, photographic reproductions, microform, electronic form (offline, online), or any other reproductions of similar nature. On Behalf of all authors AP.

## Conflict of Interest

The authors declare that the research was conducted in the absence of any commercial or financial relationships that could be construed as a potential conflict of interest.
